# Impact of Genetic Polymorphisms on the Efficacy and Safety of Isoniazid in Saudi Tuberculosis Patients

**DOI:** 10.1155/ijog/6664418

**Published:** 2025-08-23

**Authors:** Mai A. Alim A. Sattar Ahmad, Huda Mohammed Alkreathy, Ahmed Ali, Sherif Ahmed, Hala Makki

**Affiliations:** ^1^ Department of Clinical Pharmacology, Faculty of Medicine, King Abdulaziz University, Jeddah, Saudi Arabia, kau.edu.sa; ^2^ King Abdulaziz University, Faculty of Science, Biological Sciences Department, Jeddah, Saudi Arabia, kau.edu.sa; ^3^ King Abdulaziz University, Centre of Excellence for Bionanoscience Research, Jeddah, Saudi Arabia, kau.edu.sa; ^4^ King Abdulaziz University, Princess Al Jawhara Albrahim Centre of Excellence in Research of Hereditary Disorders, Jeddah, Saudi Arabia, kau.edu.sa; ^5^ Al Borg Diagnostics, R&D Department, Jeddah, Saudi Arabia; ^6^ Ain Sham University, Faculty of Agriculture, Department of Genetics, Cairo, Egypt

**Keywords:** cytochrome CYP2E1, glutathione S-transferases (GSTM1), isoniazid, N-acetyltransferase 2 (NAT2), tuberculosis

## Abstract

**Introduction:**

Responses to antitubercular drugs like isoniazid (INH) are influenced by genetic polymorphisms in metabolizing enzymes and transporters.

**Objectives:**

This study is aimed at analyzing genetic polymorphisms of NAT2, CYP2E1, and GSTM1 genes in Saudi TB patients, monitoring INH drug levels, and exploring correlations between these genetic variations, drug levels, hepatotoxicity incidence, and clinical outcomes.

**Method:**

This prospective cohort design was conducted at King Abdul‐Aziz University Hospital in Jeddah, Saudi Arabia. It followed 50 TB patients undergoing first‐line anti‐TB treatment for 6 months. Genotyping and INH serum concentration measurements were conducted.

**Results:**

The mean INH plasma drug levels measured in 30 patients were 2.86 ± 2.80. The presence or absence of the GSTM1 does not statistically affect the plasma INH level between the TB patients with no significant association between GSTM1 and clinical response, while high plasma concentration of INH was significantly associated with improved clinical response. The present study demonstrated no NAT2 and CYP2E1 gene variations in Saudi TB patients but has identified a GSTM1 variant in 68% of patients. The presence or absence of the GSTM1 gene variant appears to not affect INH drug level or clinical outcomes.

**Conclusion:**

Clinicians should consider individualized TB treatment based on genetic and demographic factors.

## 1. Introduction

Tuberculosis, a contagious disease caused by Mycobacterium tuberculosis, affects the lungs and other body organs. Its global burden is high, especially in HIV‐prevalence communities. Only 10% of immunocompetent individuals can develop TB within 2 years of exposure. HIV‐infected patients and diabetics are more prone to TB due to their immunocompetency [1, 2]. According to the World Health Organization (WHO) Annual Report 2020, nearly 10.0 million people were infected with TB in 2019; the death toll from TB was estimated to be nearly 1.2 million in HIV‐negative persons. In 2019, men aged 15 years or more comprised 56% of the population who developed TB while women accounted for 32% and children below the age of 15 accounted for 12% only [3]. The *Saudi Arabia Health Statistical Yearbook 2021* reported that the incidence of pulmonary TB showed a promising reduction from 7.95% (2505 cases per 100,000 of the population) in 2015 down to 6.62% (2264 cases per 100,000 of the population) in 2019 with high variability among Saudi Arabia regions; the highest incidence was found in Jeddah, 14.37/100,000 of the population, and Qassim was found to have the lowest incidence, 2.02/100,000 of the population. The most effective regimen recommended by WHO and the American Centers for Disease Control and Prevention includes isoniazid (INH), rifampicin (RIF), pyrazinamide (PZA), and ethambutol (EMB) 7 days/week for 8 weeks followed by INH and RIF 7 days/week for 18 weeks for newly diagnosed, drug‐susceptible pulmonary TB. The regimen differs according to the patient’s conditions and certain circumstances [4]. The continuation phase should be given for 4 months for most patients with drug‐susceptible TB. However, a 7‐month continuation phase is recommended for patients with cavitary pulmonary TB with positive sputum culture, those who received no PZA in the intensive phase, and HIV patients receiving no antiretroviral treatment. Moreover, patients receiving once‐weekly INH and RIF with positive sputum culture at the end of the intensive phase should have extended treatment regimens [4]. The second‐line antituberculous drugs are subdivided into groups: fluoroquinolones (ofloxacin, levofloxacin, moxifloxacin, and ciprofloxacin) and injectable antituberculous drugs (streptomycin, kanamycin, amikacin, and capreomycin). Less effective second‐line antituberculous drugs are ethionamide/prothionamide, cycloserine/terizidone, and p‐aminosalicylic acid. Another reason for drug discontinuation is treatment failure due to inadequate drug levels that contribute to the development of multidrug‐resistant TB (MDR‐TB). MDR‐TB requires the use of second‐line anti‐TB that is difficult to obtain and is much more expensive and toxic than first‐line anti‐TB. Therapeutic drug monitoring (TDM) is used to optimize dosing that maximizes therapeutic benefit while minimizing toxicity. It is not routinely used, and there is little evidence to guide TDM in clinical practice. TDM will allow us to identify patients with abnormal pharmacokinetics, drug interaction and noncompliance patients.

In the last decades, it became obvious that there is an association between the pharmacological response to many drugs’ efficacy, adverse effects and genetic polymorphisms of metabolizing enzymes, transporters etc. in humans. INH is a prodrug. Its first step of metabolism occurs by N‐acetyltransferase 2 (NAT2) which is well known to be under genetic control, so that fast, intermediate, or slow metabolizer has been identified. Certain major metabolites of INH were documented as the potential cause of hepatotoxicity. NAT2 polymorphisms is determined by acetylator status od slow, immediate, and fast, which influences INH plasma toxicity level, where slow acetylators exhibit highier concentration of INH increasing the risk of toxicity while fast acetylators may exhibit subtherapeutic drug levels risking treatment failure [5].

INH is initially metabolized by phase I reaction to form acetyl INH, which is hydrolyzed to acetyl hydrazine, which is proposed to be oxidized by Cytochrome P450 2E1 (CYP2E1) to hepatotoxic intermediates. Some of these hepatotoxic metabolites are detoxified by glutathione S‐transferase (GST). Therefore, polymorphism in some of these enzymes, that is, NAT2, CYP2E1, and glutathione S‐transferase M1 (GSTM1) is likely to affect the incidence and severity of hepatotoxicity of INH [5, 6]. NAT2 polymorphisms have been shown to cause individual variation in INH N‐acetylation, having the slow acetylators more susceptibility to develop hepatotoxicity than those with high NAT2 activity [7–9]. Common polymorphisms in CYP2E1 have also been shown to be associated with drug‐induced hepatotoxicity in tuberculosis patients [9, 10]. Homozygosis of GSTM1 nonfunctional (null) genotypes, which cause a lack of enzyme activity, was suggested to increase the risk for hepatotoxicity to antituberculosis drugs [11, 12].

Global research reported a high prevalence of NAT2 polymorphism, around 40%–60% of slow acetylators in the Asian, 58% from the Moroccan population while limited data were reported from the Middle East [13]. However, Saudi‐specific data remain lacking. This study seeks to address this gap by providing evidence for Saudis, providing a novel finding with implications for regional guidelines. Furthermore, CYP2E1 related polymorphism rates were also reported to be around 2.3% (CYP2E15B) in Saudi in contrast to higher rates in the Asian population (15%–20%) [14].

### 1.1. Objectives

The study is aimed at measuring serum concentrations of the anti‐TB drug INH early during treatment and to establish a correlation between polymorphism in NAT2 and CYP2E1 genes with well documented roles in INH metabolism, hepatotoxicity, and clinical outcomes with GSTM1 variants.

## 2. Materials and Methods

### 2.1. Research Design, Population, and Selection of Samples

The present study is a prospective cohort study design. The population of this study consisted of Saudi nationality patients with active TB using first‐line anti‐TB treatment confirmed diagnosis by Microbiology and x‐ray. The study took place at King Abdulaziz University Hospital (KAUH), confirming diagnosis through appropriate investigations. The sample of the research included 50 TB patients whose treatment was done according to KAUH guidelines. The choice of this sample size was motivated by the declining incidence of TB in Saudi Arabia from 2015 to 2019 and a comparable study from Morocco on NAT2 [15]. Regimen therapy consisted of first‐line antituberculous drugs, INH, RIF, EMB, and PZA for an initial 2‐month treatment phase followed by a continuation phase of either 4 or 7 months. Demographic characteristics, patients’ history, laboratory test results, and the history of the treatment were taken from the patient’s electronic medical records. All the subjects have undergone genotyping for NAT2, CYP2E1, and GSTM1 gene polymorphisms. Then, the genotypes were correlated with INH drug level by measuring C2, that is, concentration 2 h postdose (microgram per milliliter). Also, the correlation between genotypes and clinical outcomes of INH was assessed.

### 2.2. Inclusion Criteria

Male and female patients who were aged 18 and above were included. Only those with active pulmonary tuberculosis are eligible. The study also included patients receiving standard dosages of INH, RIF, EMB, and PZA as first‐line anti‐TB medications.

### 2.3. Exclusion Criteria

Patients with abnormal baseline liver function or a history of chronic liver disease; documented HIV; hepatitis B or hepatitis C virus; concurrent use of any hepatotoxic drugs, corticosteroids, immunosuppressant drugs, or herbal medicine; and extrapulmonary tuberculosis were excluded from the study. Patients who are not fully or partially drug compliant as shown in their medical records were also not included.

### 2.4. Data Collection Form

The data collection form included the following elements: Demographic information including gender, age, and weight; the patient’s daily INH dosage; and AST and ALT levels were measured as part of the baseline lab study after 6 months of INH therapy; genotyping for important NAT2, CYP2E1, and GSTM polymorphisms and INH blood levels 2 h after dosing [13] were also measured.

### 2.5. Implemented INH Protocol in KAUH

The patients with pulmonary TB at KAUH were treated according to WHO guidelines for a fixed‐dose combination regimen that included INH 300 mg once daily. Patients are followed up closely in the first 3 months to ensure compliance with the medication received. The number of tablets is counted in each visit along with arranging them in a pill organizer.

### 2.6. Analysis and Sample Collection for INH

Blood samples were withdrawn from patients for INH measurement 2 h postoral dose, using a serum‐separation tube. When the samples were received, the process started immediately by centrifuging the sample for 5 min at 3500 rpm; then, samples were stored at −80° C until analyzed. The INH ELISA kit (Catalog #E4765‐100), obtained from BioVision, was used to determine the INH concentration in human serum samples. It is a competitive‐based ELISA that can quickly determine a broad range of INH concentrations (0.2–45 *μ*g/mL).

### 2.7. Calculation

A standard calibration curve was plotted using the standard concentration on the *x*‐axis versus the average optical density value on the *y*‐axis. Linear regression analysis was performed to calculate the concentration of the sample.

### 2.8. Sample Collection for Genotyping

Blood samples were collected from each participant in an ethylenediamine tetraacetic acid (EDTA) containing tube. The blood samples were then transferred to the Centre of Excellence in Genomic Medicine Research at King Fahad Research Centre, King Abdulaziz University, Jeddah, KSA. Blood and the samples were stored at −20°C until use. Samples were genotyped by conventional polymerase chain reaction (PCR) methods.

### 2.9. Molecular Biology Techniques

#### 2.9.1. Blood DNA Extraction Procedure

DNA was extracted using the QIAGEN DNA extraction kit.

### 2.10. Primers

Primer’s sequences (Forward and Reserve) for the PCR amplification and the length of amplified products are shown in Table 1.

**Table 1:** Primers used for amplification of different deoxyribonucleic acid (DNA) regions.

**SNP**	**Forward 5** ^′^ **to 3** ^′^	**Reverse 5** ^′^ **to 3** ^′^	**Primer length**
NAT2	GCCTCAGGTGCC TTGCATTT	CGTGAGGGTAGAGAGGATAT	21
CYP2E1	CCAGTCGAGTCTACATTGTCA	TTCATTCTGTCTTCTAACTGG	21
GSTM1	GGTTGGCCAATCTACTCCCAGG	GCTCACTCAGTGTGGCAAAG	22

### 2.11. PCR Principle

The PCR is a fast and inexpensive technique used to “amplify” small segments of DNA. It involves three major steps: denaturation at 93°C–95°C, where the double‐stranded DNA is heated to separate it into single strands, providing a template for replication; annealing at 45°C–58°C to allow primers to attach to their complementary sequences on the single‐stranded DNA to allow polymerase enzymes to initiate DNA copy; and extension at 72°C to synthesize a new DNA strand by adding nucleotides to the primer, building a complementary strand to the template DNA. The ideal working temperature for the polymerase is 72°C, which allows the Taq polymerase enzyme to add nucleotides to the 3 ^′^‐end of the primer annealed to the template DNA. The resulting PCR results were considered valid by meeting the following criteria: When a product is formed, indicating successful amplification, the product is of the correct size to match the expected gene length, and the formation of only one band indicates specificity, while multiple bands are considered nonspecific binding or contamination.

### 2.12. Gel Electrophoresis Procedure

The gel electrophoresis procedure involves the preparation of TBE buffer, ethidium bromide dye, EDTA, and 2% agarose gel to check the integrity of gDNA. Reagents are prepared by mixing Tris‐base, boric acid, and EDTA in a conical flask, followed by heating in the microwave until homogenous. The gel was then placed in a tank containing 1x TBE Buffer. Gel imaging was performed using Gel Doc software, with the gel loaded into the chamber center and the UV light switched on. The camera’s focus, zoom, and aperture are adjusted to obtain the optimal image. The image was saved in the GenaTi file according to the test name and sample ID number. DNA sequencing was used to confirm a mutation reported by the referring laboratory. This method relies on the in vitro synthesis of DNA strands copied from a DNA template generated by PCR. The nascent sequencing strand was complementary to a small region of the template DNA close to the target area. Excess primers, dNTPs, and primer dimers were removed from the PCR product using a centrifugal filter. To detect newly synthesized chains, a cycle sequencing reaction was performed on the purified product in a single tube. This method involves four ddNTPs labeled with a different fluorescent dye, plus dNTPs and DNA polymerase. An extension DNA product was produced, and unincorporated dye terminators were removed by DNA precipitation. The product was analyzed using the ABI350/3500xl. The sequencing procedure involves using a control DNA template, M13mp18 as a single‐stranded control and pGEM as a double‐stranded control, and no calibrators were used. A negative control (nontemplate control) was included in the amplification to detect any contamination. A 5% polyacrylamide gel was used to approximate the amount of product to use in the sequencing reaction. All reagents passed the quality control test before use, and all working reagents were checked for availability and sufficiency. The laboratory protocol and proper technical practices were followed to reduce the risk of sample contamination by exogenous DNA and DNase. The sequencing procedure involves first purification using an Ethanol Precipitation Master Mix, which is pipetted into each PCR product tube. The tubes were then centrifuged for 30 min, washed, and dried. The PCR reaction products were loaded into a 5% polyacrylamide gel to check the reaction performance. The cycle sequencing procedure involves 0.2 tubes labeled with the GenaTi number and test identification, and the cycle sequencing master mix was prepared in a 0.2 tube using RNAse DNAse Free Water, 5X Sequencing Buffer, Big Dye Terminator V3.1 or 1.1 (RR), and Purified PCR Product. The master mix is added to each PCR tube, and the lid of the tube is closed to avoid contamination. On the thermal cycler, the preprogrammed cycle of the test was selected. The cycles were programmed as Table 2. The procedure involved a second purification, followed by a 70% ethanol wash twice. The tubes were discarded and dried overnight. The genetic analyzer was prepared by adding 15 *μ*L of formamide to each tube, loading them on a thermal cycler, and performing the’ shock effect’ on ice. A sample sheet was prepared for the ABI 3500/3500XL and started to run. The sequencing results were compared to the Genome Reference Consortium reference sequence of the Homo sapiens XYZ gene, NCBI (National Centre of Biotechnology Information), which contains databases related to life sciences. The nomenclature was written according to the Human Genome Variation Society. The sequenced sample was shown in a multiple‐peak arrangement with four different colors. The colors present green peaks for adenine bases (A), blue peaks for cytosine bases (C), black peaks for guanine bases (G), and red peaks for thymine bases (T), and the chromatogram view of the sequenced sample, along with the genetic bases aligned below the peaks, is illustrated.

**Table 2:** Cycle sequencing program.

	**Steps**	**Cycle sequencing**
1	Initial denaturation step	96°C for 1 min
2	Denature	96°C for 10 s
3	Annealing	…°C for 5 s
4	Extension	60°C for 4 min
5	Hold at	4°C

*Note:* Cycle times: denature to extension.

### 2.13. Determination of Hepatotoxicity

In this study, the plasma levels of ALT (7–56 U/L) and AST (8–48 U/L) were used to assess the incidence of hepatotoxicity. A slight increase of less than two times the normal upper limit of ALT and AST plasma levels was considered to be mild hepatotoxicity. It is considered moderate hepatotoxicity if the normal upper limit is increased between 3 and 5 times. Additionally, a patient is said to have severe hepatotoxicity if the ALT and AST are about 7 times higher than the normal upper limit.

### 2.14. Statistical Tool

The current study used Statistical Package for the Social Sciences (SPSS) software. The value of *p* is statistically significant when it is (< 0.05). The homogeneity of variance and normality of data distribution were assessed using Levene’s test and Shapiro–Wilk test, respectively. The dependent variable was normally distributed with equal variance. Hence, the data were analyzed using appropriate parametric tests. Paired *t*‐test was done to analyze the impact of INH on hepatic function. The chi‐square test was used to determine factors associated with clinical response (outcomes) among TB patients. Independent sample *t*‐test was used to analyze the effect of patients’ age, body mass index (BMI), plasma level of INH, ALT, and AST on clinical outcome. Pearson’s correlation was used to assess the relationship between hepatic function and clinical characteristics of the study group. Multivariate logistic regression was used to determine the predictor of clinical outcomes while controlling for confounding factors.

## 3. Results

### 3.1. Demographic Characteristics

In the present study, 50 INH‐treated tuberculosis patients were enrolled. Table 3 shows patients’ demographic and clinical characteristics. Most of the TB patients in this study were male (*n* = 34, 68.0%). Moreover, the average age of the patients was 42.06 ± 16.68, thus, suggesting that they were middle‐aged population. While the mean (minimum‐maximum) of patients’ BMI was 21.58 ± 5.78 (11.65 − 43.28), with about 41% being underweight (*n* = 19). The generally accepted range of normal BMI is between 18.5 and 24.9. However, the highest and lowest BMI observed were 43.28 (obese) and 11.65 (underweight), respectively. Forty‐one percent of the patients were underweight (*n* = 19). The NAT2 and CYP2E1 gene variants were not found in any patients throughout the study. More than half of the patients (*n* = 34, 68.0%) have GSTM1 gene variants. The mean plasma levels of alanine transaminase and aspartate transaminase were 20.60 ± 13.45 (7.00–60.00) and 30.60 ± 33.90 (14.00–188.00), respectively. In approximately 8% and 6% of the patients, respectively, the plasma levels of ALT and AST were abnormally high. Lastly, more than half of the study participants (*n* = 29, 58%) had improved clinical responses within the study period.

**Table 3:** Demographic and clinical characteristics of patients (*N* = 50).

**Variables**	**Frequency**	**Percent (%)**
Gender		
Male	34	68.0
Female	16	32.0
Age (years) ^∗^		
20–29	10	20.8
30–39	12	25.0
40–49	9	18.8
50–59	9	18.8
≥ 60	8	16.7
Body mass index ^∗∗^		
Underweight	19	41.3
Normal weight	18	39.1
Overweight	6	13.0
Obese	3	6.5
Presence of GSTM1		
No	16	32.0
Yes	34	68.0
Plasma level of alanine transaminase (ALT)		
Low	6	12.0
Normal	40	80.0
High	4	8.0
Plasma level of aspartate transaminase (AST)		
Low	4	8.0
Normal	43	86.0
High	3	6.0
Clinical response		
Improved	29	58.00
Not improved	21	42.00

^∗^Mean age = 42.06 ± 16.68.

^∗∗^Mean BMI = 21.57 ± 5.78.

### 3.2. Effect of INH Therapy on Liver Function

**Figure 1:**
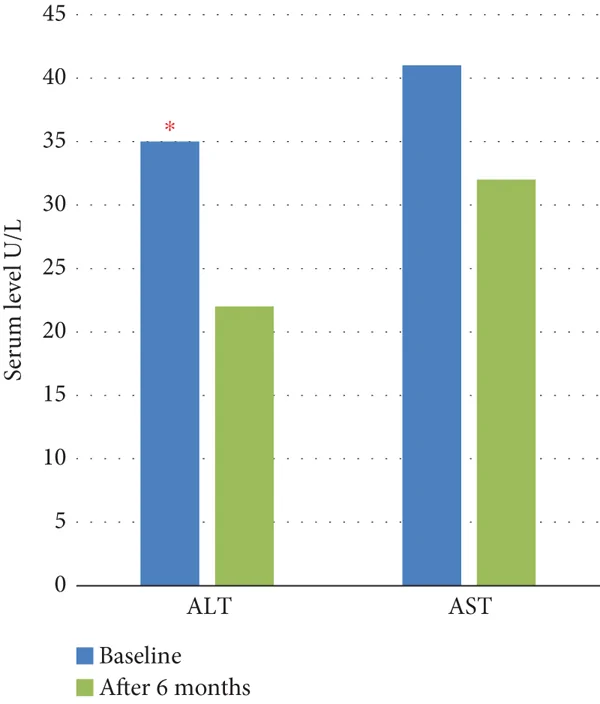
Alanine transaminase (ALT) and aspartate transaminase (AST) serum level after 6 months among tuberculosis patients. ∗Significant at *p* < 0.05.

INH drug level was measured in 30 patients with a maximum value found of 10.60 *μ*g/mL and a minimum value of 0.07 *μ*g/mL. Normal INH level is between 3 and 6 *μ*g/mL. The mean INH value was 2.86 ± 2.80. Table 4 shows the effect of INH therapy on liver function among TB patients. In this study, plasma levels of ALT and AST were the parameters used for measuring liver toxicity in TB patients. Hence, a paired sample *t*‐test was performed to assess whether there is a significant mean difference in ALT and AST plasma levels at baseline and 6 months posttreatment with INH (Figure 1). The results revealed that the mean plasma level of ALT was significantly (*p* < 0.05) higher at baseline compared to 6 months postintervention with INH therapy (35.44 ± 34.77 vs. 22.03 ± 14.44, *p* = 0.042). The mean plasma level of AST was higher among the TB patients at baseline than after 6 months of treatment with INH, but not statistically significant (41.46 ± 36.99 vs. 32.33 ± 37.38, *p* = 0.329). The present results revealed that AST levels among TB patients tend to decrease after 6 months of INH therapy, but this reduction was not statistically significant (Figure 1).

**Table 4:** Effect of isoniazid treatment for 6 months use on liver function among tuberculosis patients.

**Variables**	**Mean (SD)**	**95% CI**		**p** **value**
		**Lower**	**Upper**	
Plasma level of ALT				
Baseline	35.44 ± 34.77	0.497	26.323	0.042 ^∗^
6 months posttreatment	22.03 ± 14.44			
Plasma level of AST				
Baseline	41.46 ± 36.99	‐9.576	27.83	0.329
6 months posttreatment	32.33 ± 37.38			

*Note:* Number of patients = 50.

Abbreviations: ALT, alanine transaminase; AST, aspartate transaminase; CI: confidence interval; SD, standard deviation.

^∗^Paired *t*‐test is statistically significant at *p* < 0.05.

### 3.3. Association of Clinical Outcomes With Patients’ BMI, Age, Gender, GSTM1, Alanine Transaminase, and Aspartate Transaminase

Table 5 demonstrates the association of clinical response to INH therapy with patients’ demographic and clinical characteristics. The results demonstrate that the patient’s BMI (*X*2 = 20.749, *p* < 0.001) is significantly associated with the clinical response of TB patients on INH therapy. Most TB patients whose BMI is within the normal weight range experienced improved clinical response than those underweight or overweight, even obese. The results also show that the patient’s plasma levels of ALT (*X*2 = 7.170, *p* = 0.028) are significantly associated with the clinical response of TB patients to INH therapy. Most TB patients with normal plasma levels of ALT reported improved clinical response after taking INH therapy. Nevertheless, the gender (*X*2 = 0.030, *p* = 0.863), age (*X*2 = 0.378, *p* = 0.984), GSTM1 (*X*2 = 0.196, *p* = 0.658), and plasma level of AST (*X*2 = 3.730, *p* = 0.155) have no relationship with the clinical response of TB patients receiving INH therapy. Furthermore, the multivariate logistic regression analysis showed that BMI significantly predicted the clinical response of TB patients receiving INH (Table 6). Specifically, the finding demosntrated that TB patients with normal BMI are about 2.3 times more likely to experienced improved clinical response compared to those who are obese (OR = 2.300, 95% CI: 0.564–6.270, *p* = 0.047).

**Table 5:** Association of clinical response with patients’ demographic and clinical characteristics.

**Variables**	**Clinical response**		**X** ^2^	**p** **value**
	**Not improved**	**Improved**		
Gender			0.030	0.863
Male	14 (66.7)	20 (69.0)		
Female	7 (33.3)	9 (31.0)		
Total	21 (100.0)	29 (100.0)		
Age (years)				
20–29	4 (19.0)	6 (22.2)	0.378	0.984
30–39	6 (28.6)	6 (22.2)		
40–49	4 (19.0)	5 (18.5)		
50–59	4 (19.0)	5 (18.5)		
≥ 60	3 (14.3)	5 (18.5)		
Total	21 (100.0)	27 (100.0)		
Body mass index (kg/m^2^)				
Underweight	6 (35.3)	13 (44.8)	20.749	< 0.001 ^∗^
Normal weight	2 (11.8)	16 (55.2)		
Overweight	6 (35.3)	0 (0.0)		
Obese	3 (17.6)	0 (0.0)		
Total	17 (100.0)	29 (100.0)		
Presence of GSTM1				
No	6 (28.6)	10 (34.5)	0.196	0.658
Yes	15 (71.4)	19 (65.5)		
Total	21 (100.0)	29 (100.0)		
Plasma level of ALT				
Low	1 (4.8)	5 (17.2)	7.170	0.028 ^∗^
Normal	16 (76.2)	24 (82.8)		
High	4 (19.0)	0 (0.0)		
Total	21 (100.0)	29 (100.0)		
Plasma level of AST				
Low	0 (0.0)	4 (13.8)	3.730	0.155
Normal	19 (90.5)	24 (82.8)		
High	2 (9.5)	1 (3.4)		
Total	21 (100.0)	29 (100.0)		

*Note:* Number of patients = 50.

Abbreviations: ALT, alanine transaminase; AST, aspartate transaminase; GSTM1, glutathione S‐transferase M1.

^∗^Chi‐square test is statistically significant at *p* < 0.05.

**Table 6:** Predictors of patients’ clinical response.

**Variables**	**Odds ratio (95% C)** **Improved = 1; not improved = 0**	**p** **value**
Gender		
Male	1.067 (0.816–1.435)	0.063
Female	Reference	
Age (years)		
20–29	2.077 (0.680–3.411)	0.087
30–39	1.648 (0.584–2.151)	0.073
40–49	0.844 (0.597–1.183)	0.314
50–59	1.072 (0.760–1.828)	0.085
≥ 60	Reference	
Body mass index (kg/m^2^)		
Underweight	1.802 (1.163–2.985)	0.078
Normal weight	2.300 (0.564–6.270)	**0.047** ∗
Overweight	0.932 (0.790–1.778)	0.208
Obese	Reference	
GSTM1		
No	0.446 (0.019–1.806)	0.533
Yes	Reference	
Plasma level of ALT		
Low	2.082 (0.913–4.754)	0.081
Normal	1.646 (0.872–2.089)	0.080
High	Reference	
Plasma level of AST		
Low	0.309 (0.076–0.885)	0.351
Normal	1.102 (0.768–2.523)	0.065
High	Reference	

### 3.4. Association of Clinical Outcomes With Serum INH Level

**Figure 2:**
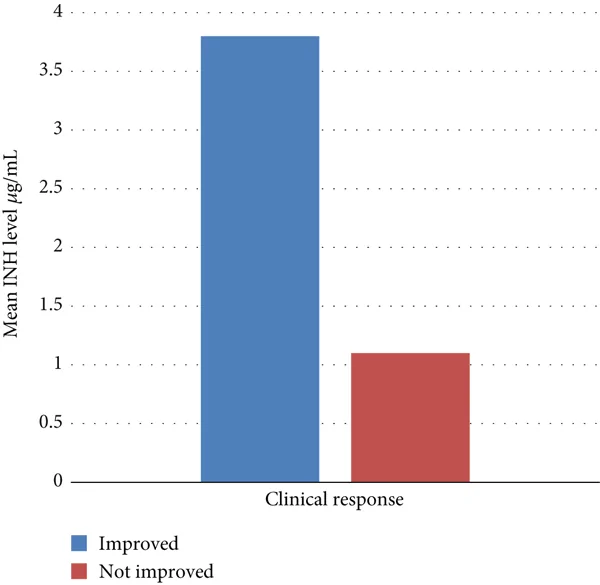
Association of clinical response with serum isoniazid level. ∗Significant at *p* < 0.05.

Patients with improved clinical symptoms had a higher concentration of INH (3.79 ± 3.00 * μ*g/mL) compared to those without improvement in clinical response (1.09 ± 1.25 * μ*g/mL), *t* = −2.702, *p* = 0.012 (Figure 2). This finding indicates that a higher INH plasma content is linked to a better clinical response to therapy. Comparing patients whose clinical symptoms improved to those whose symptoms did not, it was found that the latter group had considerably greater blood levels of INH.

### 3.5. Genetic Variation Detected by Sequencing Analysis

**Figure 3:**
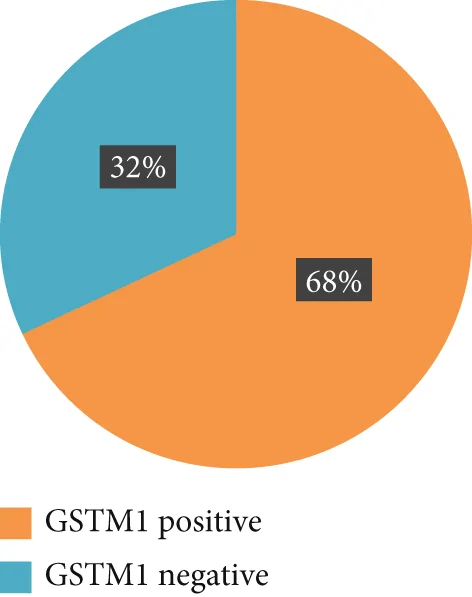
Glutathione S‐transferase M1 (*GSTM1*) percentage among Saudi tuberculosis patients.

**Figure 4:**
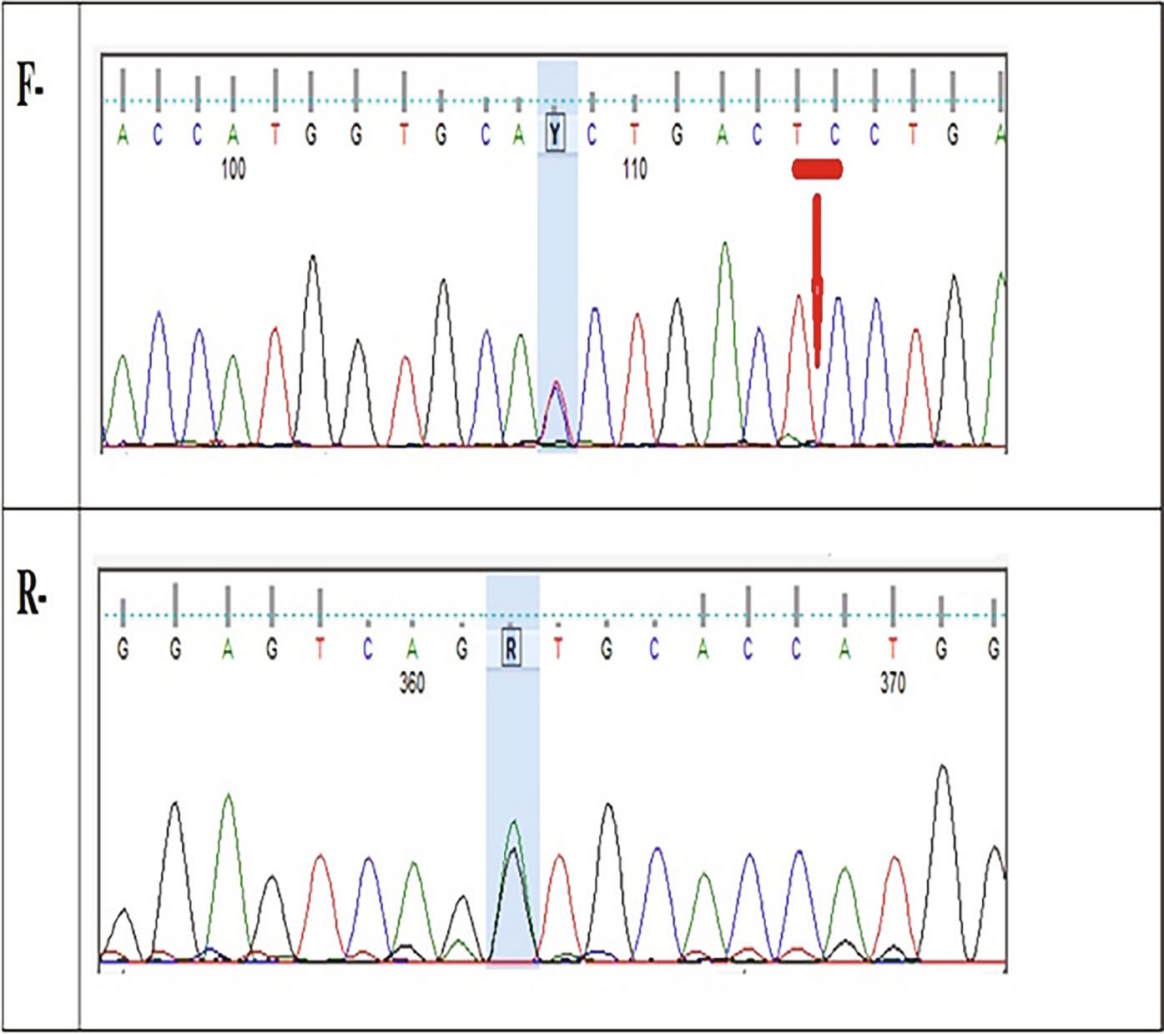
Representative sequencing of rs713040 heterozygous.

**Figure 5:**
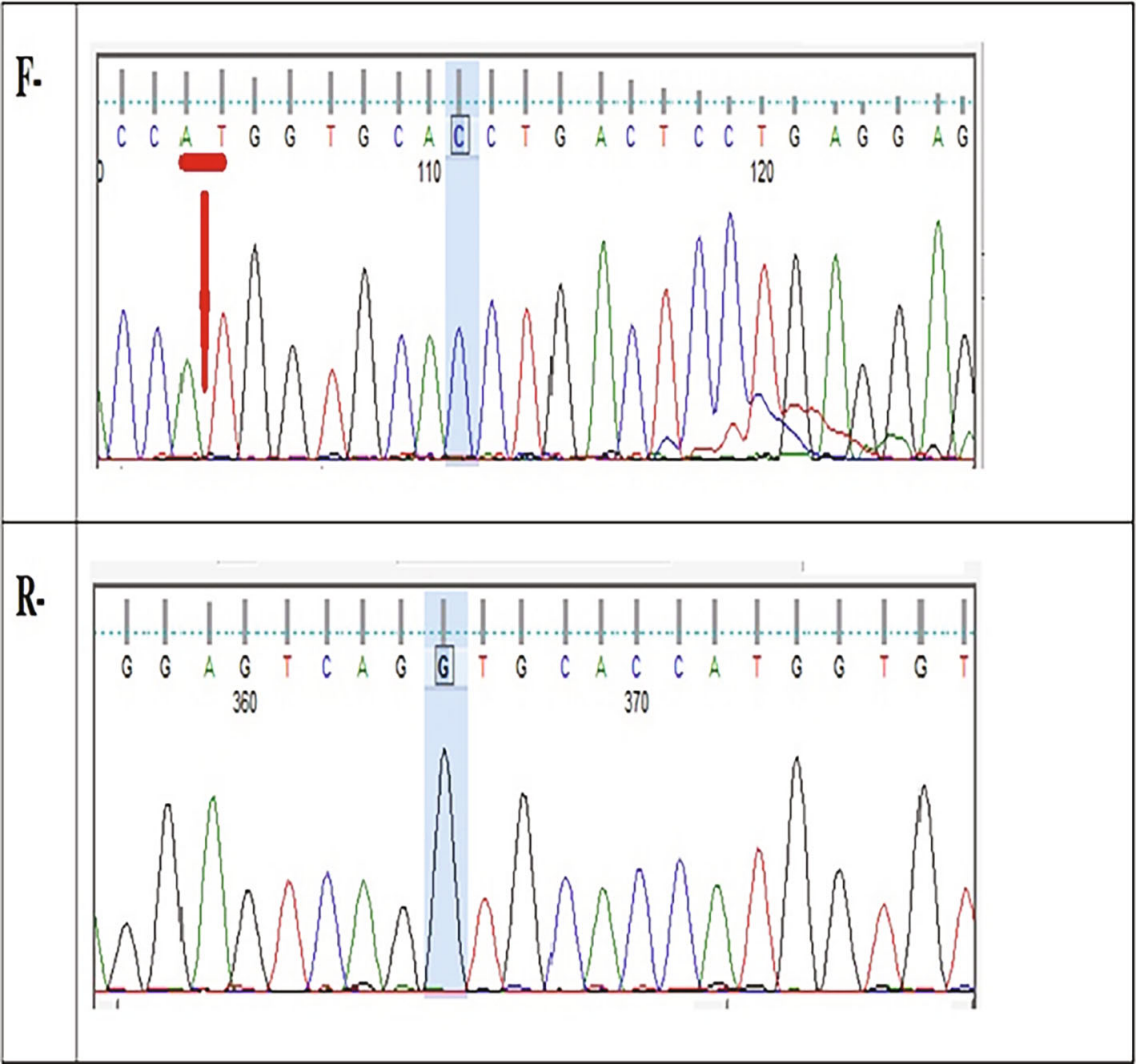
Representative sequencing of rs713040 homozygous.

**Figure 6:**
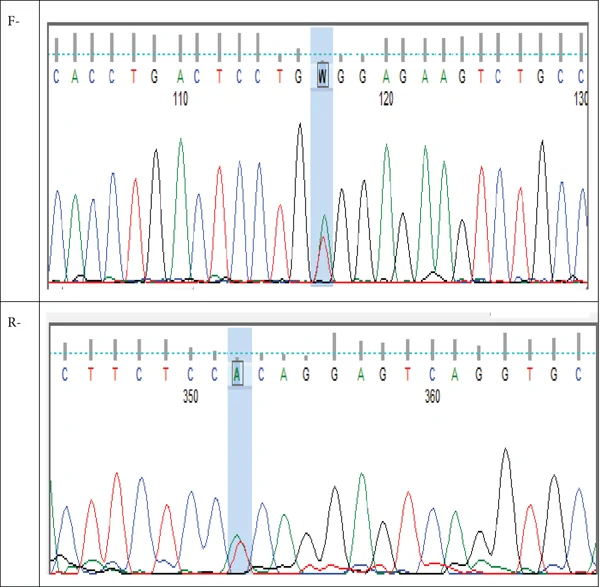
Representative sequencing of rs334 heterozygous.

**Figure 7:**
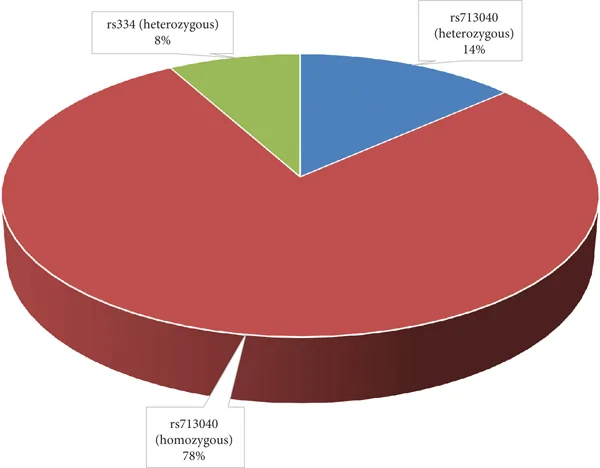
Glutathione S‐transferase M1 (*GSTM1*) mutation percentage summary.

The present results showed no NAT2 or CYP2E1 genetic variant in Saudi patients, which means that variations in these genes are not common in our Saudi patients and they are less likely to influence medication kinetics. On the other hand, the present result demonstrates that 68.0% of the patients with GSTM1 genetic polymorphism, as shown in Figure 3. The present findings revealed that the rs713040 T > C mutation was detected in 5 cases (10%) as a heterozygous (double peaks) missense mutation; the mutation is a thymine‐to‐cytosine nucleotide substitution and not detected in the control sample, as shown in Figure 4. The rs713040 T > C mutation was detected in 29 cases (58%) as homozygous (single peaks); the mutation is thymine‐to‐cytosine nucleotide substitution and not detected in the control sample, as shown in Figure 5. Finally, the rs334 A > T mutation was also detected in three cases as a heterozygous (double peaks) missense mutation; the mutation is an adenosine‐to‐thymine nucleotide substitution and not detected in the control sample, as shown in Figure 6. Summarized results of SNPs analysis have been shown in Table 7. A summary of GSTM1 mutation percentage found in our patients is shown in Figure 7. The chromatogram shows the colors present are green peaks for adenine bases (A), blue peaks for cytosine bases (C), black peaks for guanine bases (G), and red peaks for thymine bases (T). PCR products amplified with primers of rs713040 using the forward primer and reverse primer; the forward primer shows two peaks T/C in SNP position, and the reverse primer of the same sample shows two peaks A/G in SNP position.

**Table 7:** Results of single nucleotide polymorphism (SNP) analysis using sanger sequencing (*N* = 50).

	**NCB data**		**Sample**		**Position**	**Amnio acid**
	**Forward**	**Reverse**	**Forward**	**Reverse**		
rs713040 T > C	T	A	Y	R	chr11:5227013	H (His) > H (His)
rs713040 T > C	T	A	C	G		
rs334 A > T	A	T	W	W	chr11:5227002	E (Glu) > V (Val)

### 3.6. Effect of GSTM1 on Liver Function and Clinical Characteristics of Tuberculosis Patients on INH Therapy

**Figure 8:**
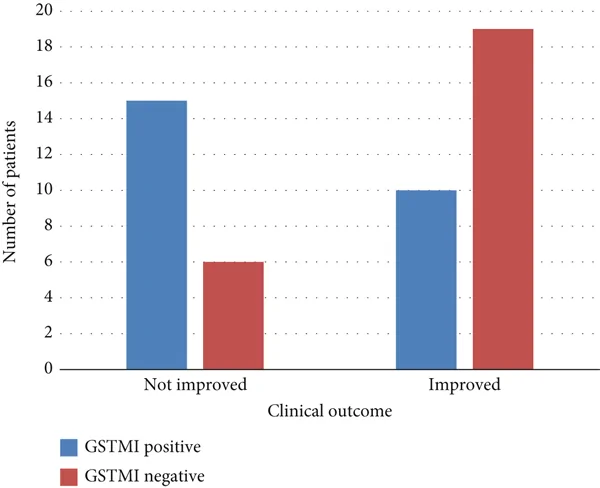
Association of clinical outcome with availability of glutathione S‐transferase M 1 (*GSTM1*) gene polymorphism.

Table 8 shows the effect of GSTM1 on liver function, clinical characteristics, and plasma drug level of TB patients on INH therapy. The present results demonstrate no significant difference in plasma ALT between TB patients with GSTM1 (19.28 ± 13.77) and those without (23.25 ± 12.80), *t* = 0.963, *p* = 0.341. Similarly, the plasma AST levels between patients with (28.72 ± 30.24) or without GSTM1 (34.38 ± 41.10) do not differ significantly, *t* = 0.541, *p* = 0.591. In other words, the plasma AST levels for patients who have GSTM1 and those who do not have the genetic variant are the same. Additionally, the results demonstrate no statistically significant difference in the plasma INH level between TB patients with GSTM1 (2.70 ± 2.91) and patients without (3.64 ± 2.55), *t* = 0.667, *p* = 0.511. Furthermore, regarding the relationship between GSTM1 and clinical characteristics, patients’ age (43.87 ± 18.56 vs. 41.21 ± 15.94, *t* = 0.524, *p* = 0.560) and BMI (21.83 ± 4.53 vs. 21.45 ± 6.37, *t* = 0.204, *p* = 0.839) have no statistically significant relationship with GSTM1. This finding implies that TB patient’s age and BMI are unrelated to the presence or absence of the GSTM1 gene variant. Yet, GSTM1 has no relationship with the clinical response of TB patients receiving INH therapy (Figure 8). The findings of the current study showed no significant association between GSTM1 and clinical response.

**Table 8:** Effect of glutathione S‐transferase M1 *(GSTM1)* on hepatic function, clinical characteristics, and plasma drug level of TB patients on isoniazid therapy.

**Variables**	**Mean (SD)**	**95% CI**		**p** **value**
		**Lower**	**Upper**	
Age (years)				
No	43.87 ± 18.56	−7.57	12.91	0.560
Yes	41.21 ± 15.94			
BMI (kg/m^2^)				
No	21.83 ± 4.53	−3.33	4.08	0.839
Yes	21.45 ± 6.37			
Plasma ALT level (6 months posttreatment)				
No	23.25 ± 12.80	−4.33	12.26	0.341
Yes	19.28 ± 13.77			
Plasma AST level (6 months posttreatment)				
No	34.38 ± 41.10	−15.39	26.71	0.591
Yes	28.72 ± 30.24			
Plasma level of INH (*μ*g/mL)				
No	3.64 ± 2.55	−1.95	3.82	0.511
Yes	2.70 ± 2.91			

*Note:* Number of patients = 50; presence of hepatotoxicity = yes/no.

Abbreviations: ALT, alanine transaminase; AST, aspartate transaminase; BMI, body mass index; CI, confidence interval; GSTM1, glutathione S‐transferase M1; INH, isoniazid; SD, standard deviation.

### 3.7. Determination of Clinical Response in Tuberculosis Patients Treated With INH

**Figure 9:**
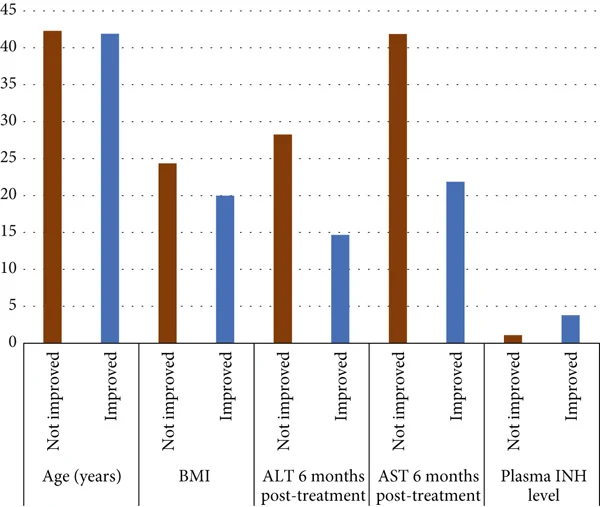
Analysis of clinical response among tuberculosis (TB) patients.

Table 9 shows the effect of patients’ age, BMI, ALT, AST, and INH plasma levels on clinical response. The findings demonstrate that TB patients who experienced improved clinical response after taking INH therapy have significantly lower BMI (19.96 ± 2.99 kg/m^2^) than those who did not report any clinical improvement (24.33 ± 8.09 kg/m^2^), *t* = 2.636, *p* = 0.012. The BMI values of patients who responded effectively to INH medication and demonstrated improved clinical response were considerably lower. Also, the findings show that patients with improved clinical outcomes had significantly lesser plasma ALT levels (14.67 ± 7.75 units) compared to those without improvement in clinical symptoms (28.24 ± 15.44 units), *t* = 3.977, *p* < 0.001. In like manner, patients who experienced better clinical outcomes had considerably lower levels of plasma AST (21.85 ± 7.07 units) than those without any clinical improvement (41.86 ± 48.98 units), *t* = 2.100, *p* = 0.041. The summary of these findings is represented in Figure 9.

**Table 9:** Mean difference analysis of clinical response among tuberculosis (TB) patients treated with isoniazid.

**Variables**	**Mean (SD)**	**95% CI**		**p** **value**
		**Lower**	**Upper**	
Age (years)				
Not improved	42.28 ± 18.21	−9.31	10.09	0.936
Improved	41.89 ± 15.81			
BMI (kg/m^2^)				
Not improved	24.33 ± 8.09	0.09	8.65	0.012 ^∗^
Improved	19.96 ± 2.99			
ALT (6 months posttreatment)				
Not improved	28.24 ± 15.44	6.70	20.44	< 0.001 ^∗^
Improved	14.67 ± 7.75			
AST (6 months posttreatment)				
Not improved	41.86 ± 48.98	0.83	39.17	0.041 ^∗^
Improved	21.85 ± 7.07			
Plasma level of INH (*μ*g/mL)				
Not improved	1.09 ± 1.25	−4.75	−0.65	0.012 ^∗^
Improved	3.79 ± 3.00			

*Note:* Number of patients = 50.

Abbreviations: ALT, alanine transaminase; AST, aspartate transaminase; BMI, body mass index; INH, isoniazid; SD, standard deviation.

^∗^ *T*‐test is statistically significant at *p* < 0.05.

### 3.8. Correlation Between Hepatic Function and Clinical Characteristics in INH‐Treated Tuberculosis Patients

Table 10 illustrates the correlation between hepatic functioning and clinical characteristics of TB patients receiving INH therapy. The findings obtained revealed that patients’ age had a significantly moderate positive correlation with the plasma AST levels (*r* = 0.437, *p* < 0.01). In other words, as TB patients’ age increases, AST plasma levels will also increase. The findings also demonstrate a moderate positive relationship between patients’ age and BMI values (*r* = 0.393, *p* < 0.01). Additionally, there is a moderate positive relationship between the plasma levels of ALT and AST (*r* = 0.331, *p* < 0.05).

**Table 10:** Relationship between hepatic function and clinical characteristics of patients with TB on isoniazid.

	**Age (years)**	**Plasma level of ALT (6 months posttreatment)**	**Plasma level of AST (6 months posttreatment)**	**Plasma level of INH (ug/mL)**	**Body mass index**
Age (years)	1	0.160	0.437 ^∗∗^	0.063	0.393 ^∗∗^
Plasma level of ALT (6 months post‐treatment)		1	0.331 ^∗^	‐0.195	0.071
Plasma level of AST (6 months post‐treatment)			1	‐0.187	0.169
Plasma level of INH (*μ*g/mL)				1	‐0.166
Body Mass Index					1

*Note:* Number of patients = 50.

Abbreviations: ALT, alanine transaminase; AST, aspartate transaminase; INH, isoniazid.

^∗∗^Correlation is significant at *p* < 0.01.

^∗^Correlation is significant at *p* < 0.05.

## 4. Discussion

INH is a commonly used antituberculosis drug despite its well‐known hepatotoxicity profile. This is because it is less expensive and has a high potency against mycobacteria. Therefore, this study sought to examine genetic polymorphism (NAT2, GSTM1, and CYP2E1) and its relationship with INH drug level, incidence of hepatotoxicity, and clinical outcomes among TB Saudi patients. In the current study, the majority of the TB patients were male (*n* = 34, 68.0%). This finding is similar to a previous study in India, where about 54% of the TB patients in the study were male [14]. In like manner, another study in Africa documented that female TB patients accounted for 41.5% out of the 142 participants in the study [15]. Therefore, this finding may simply suggest that TB is more common in men compared to women. This observation may probably be attributed to differences in lifestyle, occupation, and hormonal composition that affect the immune response. For instance, men appear to take up riskier jobs than women. The average age of the patients in the current study was 42.06 ± 16.68, thus suggesting that they were a middle‐aged population. This finding is supported by many authors that the average age of TB patients was 32.20 ± 7.41 years [14]. Another study conducted by Rana et al. reported a mean age of 43.6 ± 18.7 years [16]. Similarly, Singla et al. documented an average age of 38.00 ± 6.62 years among TB patients [17]. Another study in Morocco reported a mean age of 42.6 ± 17.7 [15]. This finding suggests that TB is more prevalent in the young middle‐aged population. The BMI findings of this study support the outcome of a previous study in Ethiopia, where about 62.1% of TB patients had a BMI within the underweight category [18]. The loss of appetite and increased metabolic rate often experienced among TB patients are likely to be responsible for the high prevalence of the underweight category observed in the current study. Patients with TB who are underweight may have weakened immune systems, which could make them more susceptible to contracting TB. In the current work, patients with improved clinical symptoms had a higher plasma concentration of INH (3.79 ± 3.00 * μ*g/mL) compared to those without improvement in clinical response (1.09 ± 1.25 * μ*g/mL). An observational and experimental study in India reported that the INH plasma level of 36 TB patients after 1 week of administration ranged from 1.17 to 4.10 *μ*g/mL, while the mean was 2.33 ± 0.88. Moreover, 16 patients in the study had INH plasma levels ranging from 2.5 to 3.5 *μ*g/mL [19]. A similar finding was also reported in a study among TB patients with diabetes mellitus who were undergoing TDM for INH and RIF in the United States. The study reported INH plasma concentration that ranged from 2.13 to 3.63 *μ*g/mL. However, for slow responders, the study reported a plasma concentration of 3.1–4.2 *μ*g/mL. Generally, the normal plasma level of INH ranges from 3 to 6 *μ*g/mL. The association between higher INH concentrations and improved clinical symptoms suggests that achieving and maintaining adequate drug levels are crucial for the effectiveness of INH in treating TB. However, patients who do not respond to treatment may benefit from interventions to enhance medication adherence. Monitoring for potential side effects or adverse reactions is essential, as increased drug concentrations may increase the risk of adverse events. The NAT2 and CYP2E1 gene variants have not been found in any patients throughout the study. A study conducted in western Saudi Arabia showed that the genotype distribution of CYP2E1∗5B and ∗6 polymorphisms was lower than the global average [20]. However, most of the TB patients in the current study had the gene, GSTM1. In contrast, a Peruvian study among 377 TB patients reported that the NAT2 gene was found in 47.48% of slow acetylators, 37.67% of intermediate acetylators, and 14.85% of fast acetylators. In Colombia, the prevalence of the NAT2 gene was found to be 37%, while approximately 50% of the Chinese and Taiwanese TB populations were estimated to have NAT2 and CYP2E1 [21, 22]. The NAT2 and CYP2E1 polymorphism has been identified in the literature as a marker of hepatotoxicity induced by anti‐TB medications, including INH. The NAT2 gene is reported to encode for the slow phenotype which predisposes TB patients to an increased risk of developing anti‐TB‐induced liver injury [23]. The absence of NAT2 and CYP2E1 genes in the current study may be attributed to the small sample size of the study group. The absence of CYP2E1 and NAT2 genes may suggest that TB patients in this study have limited ability to metabolize INH, thus leading to sustained elevated plasma levels of the drug, which may increase the risk of experiencing adverse drug reactions. However, GSTM1 homozygous frequency was found to be 58% of the total number of treated patients in the present study. It has been reported that homozygous deletion frequencies are greater in Pacific Islander, Malaysian, Chinese, Korean, and Japanese populations [13]. Also, a few ethnic populations in India had high frequencies of GSTM1 homozygous deletion genotypes [24]. In the current study, INH drug level was measured in 30 patients with a maximum value of 10.60 and a minimum value of 0.07. The mean INH value was 2.86 ± 2.80. Following the present results, a study conducted for Korean patients found a similar mean of INH (2.8 ± 1.4), which is considered a low INH level [25]. Most studies defined normal INH levels to be between 3 and 6 *μ*g/mL. The results revealed that the mean plasma level of ALT is significantly higher at baseline compared to 6 months postintervention with INH therapy. The mean plasma level of AST was higher among the TB patients at baseline than after 6 months of treatment with INH but was not statistically significant. In contrast to the finding of this study, a Brazilian study documented a decrease in the average plasma ALT level of TB patients after 3 months and 6 months of treatment with INH. Specifically, the study found that the mean plasma ALT level was increased from 25.00 ± 13.30 to 49.93 ± 15.85 * μ*L/mL after 30 days of treatment with INH for TB patients who developed hepatotoxicity. The plasma ALT level also increased further to 82.70 ± 17.30 * μ*L/mL after 60 days of treatment. However, the plasma ALT level reduced from 23.46 ± 13.45 to 22.70 ± 2.00 * μ*L/mL after INH 2 months of treatment in patients who had not experienced liver toxicity [26]. The present results demonstrate that the patient’s BMI is significantly associated with the clinical response of TB patients on INH therapy. Most TB patients whose BMI is within the normal weight range experienced improved clinical response than those underweight or overweight, even obese. A similar finding was reported in a clinical trial among Swedish patients with rheumatoid arthritis. In that study, patients with a BMI within the normal range had better clinical outcomes than those who were obese [27]. Another study among Taiwanese TB patients found that poorer clinical outcomes were associated with TB patients who are underweight compared to those with normal body weight [28]. Additionally, low body weight was identified as a predictor of unfavorable clinical outcomes in TB patients on a thrice‐weekly antituberculosis regimen in India [29]. Tuberculosis treatment depends on a patient’s BMI, with underweight patients having compromised immune systems. Nutritional support is crucial for underweight patients, while weight management is essential for overweight or obese patients [30]. Regular liver function monitoring is essential for managing INH‐related liver toxicity, with further research needed to understand mechanisms and potential shorter INH courses for TB patients. TB patients with elevated ALT levels may require closer medication management and potentially shorter courses of INH to reduce the risk of liver toxicity. Nevertheless, gender, age, and GSTM1 gene variants have no relationship with the clinical response of TB patients receiving INH therapy. Unlike the present study, gender was associated with clinical outcomes among TB patients receiving treatment. In that study, clinical outcomes were similar for both men and women, except for male TB patients with the presence of cavities, in which clinical outcomes were comparatively lower [31]. Another study found a connection between male gender and worse clinical outcomes in TB treatment [32]. On the other hand, previous studies established a link between older or advanced age and unfavorable clinical response among TB patients on antituberculosis treatment [31, 32]. The effect of GSTM1 on clinical outcomes among TB patients is inconsequential based on available evidence. For example, the presence of GSTM1 gene variants, according to a previous study, was associated with improved clinical outcomes in TB patients [33]. However, similar to the current study finding, another previous study found no evidence of an association between GSTM1 and how TB patients respond to treatment [34]. These findings imply that INH therapy can be applied consistently across different demographic groups without the need for gender, age, and GSTM1 gene‐specific adjustments. The results of the present investigation demonstrate no significant difference in plasma ALT between TB patients who have GSTM1 and those who do not. This finding suggests that the presence or absence of GSTM1 does not appear to influence the plasma ALT levels significantly. Similarly, the plasma AST levels between patients with or without GSTM1 do not differ significantly. Contrary to research linking GSTM1‐null genotypes to hepatotoxicity in Europeans [12], there were no observed associations in Saudi patients due to population‐specific genetic or environmental modifiers. High prevalence of GSTM1 variants at 68% in this cohort, paired with a lower BMI at mean + 21.58 and minimal alcohol use, may suggest less oxidative stress, hence reducing GSTM1’s clinical relevance in this study. These differences highlight the need for ethnicity‐specific pharmacogenomic guidelines [35]. However, a study in Pakistan found no relationship between GSTM1 and hepatotoxicity [36]. In like manner, another study found no link between GSTM1 and incidence of hepatotoxicity [37]. These findings infer that other factors may significantly contribute to the issue of hepatotoxicity experienced in some patients on INH therapy. The study found no significant difference in plasma INH levels between TB patients with GSTM1 and those without, suggesting that the exact effect of GSTM1 on INH plasma concentration is unclear. Some studies suggest GSTM1 increases antituberculosis toxicity risk, while others find no relationship [34, 35, 38, 39]. The genetic variant represented by GSTM1 has little effect on the body’s ability to metabolize or absorb INH. This suggests that typical INH‐based TB therapies may benefit both groups regardless of GSTM1 variant presence in a clinical setting. Treatments based on INH should be delivered consistently regardless of GSTM1 presence. Most patients showed a benign variant rs713040 T > C mutation type which is clinically benign and represents no changes in coded amino acid (histidine) and that could explain why it does not affect INH [35]. Further, patients’ age and BMI have no statistically significant association with GSTM1. In contrast to the study finding, a study among young people in Saudi Arabia reported significantly higher BMI and body weight among people with the GSTM1 gene variant [40]. The study found that TB patients with improved clinical response after INH therapy had significantly lower BMI compared to those without improvement, which is consistent with previous research on BMI’s impact on TB patient outcomes [27, 28]. The study found that patients with lower BMIs were more likely to respond effectively to INH medication and show improved clinical response, possibly due to a correlation between lower BMI and better prognosis for TB treatment with INH, and lower plasma ALT and AST levels. This finding agrees with the outcome of a previous study which reported that TB patients with lower levels of plasma ALT and AST had significantly better clinical responses [30]. This finding proposes that people with lower ALT levels have a lesser degree of hepatic inflammation or damage, which may account for the reason why they respond to therapy better and have more favorable clinical outcomes.

### 4.1. Limitation of the Study

Limitations of the study on genetic polymorphism, INH drug levels, hepatotoxicity, and clinical outcomes in Saudi TB patients include a relatively small sample size of 50 patients; while pragmatic, it is difficult to give a robust pharmakinetic modeling, necessitating a larger sample size for more robust and generalizable findings. Though the sample size of 50 may be sufficient for detecting the prevalence of GSTM1 effects, it was underpowered to evaluate rare NAT2/CYP2E1 variants. While the study identified genetic variations in the GSTM1 genes, the genetic diversity within the population may affect drug responses differently. Confounders such as dietary habits, renal function (GFR), and epigenetic factors were not evaluated, which may influence INH metabolism, warranting further exploration of other genetic variations. The one‐time collection of a blood sample at 2 h and a 6‐month follow‐up period may not capture thorough pharmacokinetic and long‐term effects of INH, suggesting the need for multiple time‐point analysis and longer follow‐up periods to understand treatment sustainability. Additionally, the lack of comparative groups with different genetic profiles or treatment regimens hinders a comprehensive understanding of genetic polymorphisms’ specific impact on treatment responses. The study also did not consider potential confounding factors like concomitant medications or lifestyle factors, and its focus on Saudi TB patients limits generalizability to other populations, necessitating replication studies in diverse populations for validation.

## 5. Conclusion

The study identified genetic polymorphisms in GSTM1 but not in NAT2 and CYP2E1 genes among Saudi TB patients, with some patients showing specific SNPs suggesting potential variability in medication response. Most patients had lower than normal INH plasma drug levels, but improved clinical response was associated with higher drug levels and normal liver enzyme levels. The study highlights the importance of personalized treatment based on genetic and demographic factors and suggests further research for tailored TB treatment approaches.

Larger studies are needed to understand the prevalence of NAT2 and CYP2E1 genes in Saudi Arabian TB patients and to recommend routine genetic profiling and TDM. This is crucial for customizing treatment regimens, especially in high‐risk and malnourished patients. Healthcare providers should undergo training on interpreting genetic profiling, emphasizing individualized treatment strategies, and promoting medication adherence, particularly for individuals with lower INH values.

## Ethics Statement

The study was approved by the research ethics committee at King Abdulaziz University Hospital (KAUH) on October 20, 2021, with Reference Number: 496‐21. An informed consent form was signed by all participants after they were provided with verbal and written information concerning the research study. The authors confirm that the study did not involve any violation for the patients’ rights. We also confirm that this research project was conducted as per the Declaration of Helsinki.

## Consent

The participants of this study have granted consent to publish their information in a biomedical journal.

## Conflicts of Interest

The authors declare no conflicts of interest.

## Funding

This project was funded by the Deanship of Scientific Research (DSR) at King Abdulaziz University, Jeddah, under grant number G:184‐248‐1441. The authors, therefore, acknowledge with thanks the DSR for technical and financial support. [Correction added on 13 September 2026, after first online publication: The Funding statement has been updated in this version.]

## Data Availability

The datasets generated and/or analyzed during the current study are available from the corresponding author upon reasonable request.
