# Correction to “Impact of Genetic Polymorphisms on the Efficacy and Safety of Isoniazid in Saudi Tuberculosis Patients”

**DOI:** 10.1155/ijog/9865175

**Published:** 2026-09-20

Mai A. Alim A. Sattar Ahmad, Huda Mohammed Alkreathy, Ahmed Ali, Sherif Ahmed, Hala Makki, “Impact of Genetic Polymorphisms on the Efficacy and Safety of Isoniazid in Saudi Tuberculosis Patients,” *International Journal of Genomics* 2025 (2025): 6664418, https://doi.org/10.1155/ijog/6664418.

In the above‐mentioned article, affiliations number 2 and 8 were included by error. These should be removed, and as such, authors Ahmed Ali and Hala Makki should be affiliated with affiliation 1. Due to these changes, the affiliations of author Sherif Ahmed have subsequently been renumbered.

The incorrect affiliation list is as follows:

Mai A. Alim A. Sattar Ahmad,1 Huda Mohammed Alkreathy,1 Ahmed Ali,2 Sherif Ahmed,3,4,5,6,7 and Hala Makki8

1 Department of Clinical Pharmacology, Faculty of Medicine, King Abdulaziz University, Jeddah, Saudi Arabia

2 Pharmacology Department, Faculty of Medicine, King Abdulaziz University, Jeddah, Saudi Arabia.

3 King Abdulaziz University, Faculty of Science, Biological Sciences Department, Jeddah, Saudi Arabia

4 King Abdulaziz University, Centre of Excellence for Bionanoscience Research, Jeddah, Saudi Arabia

5 King Abdulaziz University, Princess Al Jawhara Albrahim Centre of Excellence in Research of Hereditary Disorders, Jeddah, Saudi Arabia

6 Al Borg Diagnostics, R&D Department, Jeddah, Saudi Arabia

7 Ain Sham University, Faculty of Agriculture, Department of Genetics, Cairo, Egypt

8 Genomics and Biotechnology Department, Faculty of Science, King Abdulaziz University, Jeddah, Saudi Arabia

The corrected affiliation list and author affiliations are shown below:

Mai A. Alim A. Sattar Ahmad1, Huda Mohammed Alkreathy1, Ahmed Ali1, Sherif Ahmed2,3,4,5,6, Hala Makki1

1 Department of Clinical Pharmacology, Faculty of Medicine, King Abdulaziz University, Jeddah, Saudi Arabia

2 Department of Biological Sciences, Faculty of Science, King Abdulaziz University, Jeddah, Saudi Arabia

3 Centre of Excellence for Bionanoscience Research, King Abdulaziz University, Jeddah, Saudi Arabia

4 Princess Al‐Jawhara Al‐brahim Centre of Excellence in Research of Hereditary Disorders, King Abdulaziz University, Jeddah, Saudi Arabia

5 R&D Department, Al Borg Diagnostics, Jeddah, Saudi Arabia

6 Department of Genetics, Faculty of Agriculture, Ain Shams University, Cairo, Egypt

Additionally, there was an error in the Funding section. The text “This research project was funded by the King Abdulaziz University (10.13039/501100004054) (G: 184‐248‐1441)” was incorrect. The corrected section is shown below:

**Funding**

This project was funded by the Deanship of Scientific Research (DSR) at King Abdulaziz University, Jeddah, under Grant Number G:184‐248‐1441. The authors, therefore, acknowledge with thanks the DSR for its technical and financial support.

These errors have been corrected in the online version of the article.

We apologize for these error.

